# Bone mineral density in elite adolescent female figure skaters

**DOI:** 10.1186/1550-2783-9-57

**Published:** 2012-12-27

**Authors:** Kathy Prelack, Johanna Dwyer, Paula Ziegler, Joseph J Kehayias

**Affiliations:** 1 Clinical Nutrition, Shriners, Hospitals For Children, 51 Blossom Street, Boston, MA, 02114, USA; 2 Department of Surgery, Massachusetts General Hospital, 51 Blossom Street, Boston, MA, 02114, USA; 3 Friedman School of Nutrition Science and Policy, Tufts University, 150 Harrison Avenue, Boston, MA, 02111, USA; 4 Frances Stern Nutrition Center, Tufts Medical Center, 800 Washington Street, Box #783, Boston, MA, 02111, USA; 5 Jean Mayer Human Nutrition Research Center on Aging, Tufts University, 711 Washington Street, Boston, MA, 02111, USA; 6 School of Medicine, Tufts University, 136 Harrison Avenue, Boston, MA, 02111, USA; 7 College of Saint Elizabeth, 2 Convent Road, Morristown, NJ, 07960, USA; 8 Body Composition Laboratory USDA Human Nutrition Research Center on Aging at Tufts University, 711 Washington Street, Boston, MA, 02111, USA

## Abstract

**Abstract:**

Elite adolescent figure skaters must accommodate both the physical demands of competitive training and the accelerated rate of bone growth that is associated with adolescence, in this sport that emphasizes leanness. Although, these athletes apparently have sufficient osteogenic stimuli to mitigate the effects of possible low energy availability on bone health, the extent or magnitude of bone accrual also varies with training effects, which differ among skater disciplines.

**Purpose:**

We studied differences in total and regional bone mineral density in 36 nationally ranked skaters among 3 skater disciplines: single, pairs, and dancers.

**Methods:**

Bone mineral density (BMD) of the total body and its regions was measured by dual energy x-ray absorptiometry (DXA). Values for total body, spine, pelvis and leg were entered into a statistical mixed regression model to identify the effect of skater discipline on bone mineralization while controlling for energy, vitamin D, and calcium intake.

**Results:**

The skaters had a mean body mass index of 19.8 ± 2.1 and % fat mass of 19.2 ± 5.8. After controlling for dietary intakes of energy, calcium, and vitamin D, there was a significant relationship between skater discipline and BMD (p = 0.002), with single skaters having greater BMD in the total body, legs, and pelvis than ice dancers (p < 0.001). Pair skaters had greater pelvic BMD than ice dancers (p = 0.001).

**Conclusions:**

Single and pair skaters have greater BMD than ice dancers. The osteogenic effect of physical training is most apparent in single skaters, particularly in the bone loading sites of the leg and pelvis.

## Introduction

Competitive figure skating is a sport that can be beneficial to bone health and the prevention of osteoporosis in female athletes. Elite female skaters, who often begin before puberty, practice up to 30 hours per week on and off the ice. Their training sessions consist of repetitive, high impact, bone loading activities, which favor bone accretion [1-3]. However competitive figure skating is also a sport which emphasizes leanness for performance enhancement and aesthetic reasons [4]. A decrease in energy availability due to intense physical activity and calorie restriction may lead to amenorrhea, bone demineralization, and stress fractures in these female athletes. [5,6] Adolescent skaters, who attain elite status, may find it particularly challenging to maintain intake adequate to support bone growth while controlling their body weight.

There are several different disciplines in figure skating, including single and pair skating, and ice dancing. Technical requirements differ among these three disciplines. For example, the required elements for female singles short program include at least three jump series that contain double and triple jumps, and jump combinations. Pair skaters have fewer required jumps, however they must incorporate at least one throw jump. So while the routines of single and pair skaters differ in their jump routines, both involve a good deal of bone loading. Ice dancers incorporate more lifts in their routines, but they execute fewer jumps then single and pair skaters. Their landing forces and mechanical bone loading are expected to be much less. We studied the differences in total and region specific bone mineral density in 36 elite, adolescent female skaters, training to compete in single, pair, or ice dancing categories. We hypothesize that BMD is greater in single and pair skaters than in their dancer counterparts.

## Methods

### Subjects

Data collected from 36 nationally ranked adolescent female figure skaters who attended a spring research camp at the US Olympic Trainer Center in Colorado Springs, CO from 1998–1999 were used for this analysis. Approval for conducting the study was received from the Human Subject Review Committee at the US Olympic Trainer Center, and from the Human Investigation Review Committee at the Tufts Medical Center in Boston. All patients provided informed consent prior to enrollment into the study.

### Assessment of dietary intake and physical activity

Prior to their arrival at the training camp, food records and detailed instructions on how to fill them out were sent to the skaters. Skaters were asked to provide 3-day dietary intake records (2 consecutive days and 1 weekend day) during the 2 months prior to their arrival at camp. Physical activity records were also collected on the same three days that participants recorded their dietary intakes to estimate physical activity level during a period of active training. During camp, information about their dietary intakes and physical activity was reviewed with the skater by study staff to clarify any issues on the record. Dietary intakes were verified, coded, entered and analyzed by a registered dietitian on the study staff using Nutritionist IV version 4.1 (First Data Bank, Inc, San Bruno, CA, 1997). Estimated intakes of calories, vitamin D, and calcium were obtained for this analysis.

### Body composition

#### Dual energy photon absorptiometry (DXA)

Bone density and body composition (lean body mass, fat mass) were determined for the whole body and specific regions using dual energy x-ray absorptiometry (DXA) with a Lunar Densitometer DPX-L Radiation (Madison,WI). Scans were conducted by individuals trained and certified in DXA use. For the scan, the participant was positioned on her back with her body straight, arms at sides, palms down, separated from thighs. Participants were scanned in the morning. Total scan time was between 11–15 minutes. Bone mineral density (BMD) for the total body (TB) and partitioned regions of the body: head, arms, legs, trunk, ribs, pelvis, and spine was determined. Specific sites of interest such as leg (L), spine (S), and pelvis (P) were selected based on their sensitivity to weight bearing bone loading and because we had reference data on that particular instrument for those specific sites available for calculation of z scores. BMD was expressed as grams per centimeter squared (gm/c^2^). Standardized scores based on age and weight matched controls as generated by the machine’s software (version 1.34; Lunar Corporation, DPX-L technical manual, Appendix C) were used in the analysis. Body composition analysis by DXA was also used to obtain % body fat on the participants.

#### Height and weight

Prior to DXA scanning, height (to the nearest 0.5 cm) using a stadiometer and weight (to the nearest 50 gms) were measured using a beam balance scale with a non-detachable weight. Measurements were taken in the morning and before training, with subjects dressed in light clothing. Body-mass index (BMI) values were then calculated as the ratio of weight (kg) to height (m) squared (kg/m^2^).

#### Data analysis

Statistical analysis was performed by using The SAS® System version 8.2 (SAS Institute Inc, Cary, NC). The relationships between skater discipline (single, pair, and dancer) and BMD standardized z scores for total body, spine, pelvis, and legs were tested using a mixed regression model while controlling for dietary intake of calories, vitamin D, calcium, BMI, and % body fat. Briefly, a model was created for each BMD density variable (total, spine, pelvic, and leg), using these BMD variables as the dependent variable, and skater discipline, dietary intakes for energy, calcium, and vitamin D, a BMI, and % body fat as the independent variables. Significant predictors were identified by the model using a significance of p < 0.05. For those continuous variables (energy, vitamin D, calcium, BMI, and % body fat) that were noted to be significant predictors, linear regression was used for comparisons to all BMD z-scores. For categorical variables (skater type) a one-way analysis of variance (ANOVA) was used to test for mean differences between the 3 skater disciplines for each BMD variable. For comparisons among groups when significance was found, a Tukey post hoc was applied. A probability (p) value of less than 0.05 was considered statistically significant. ANOVA was also used to describe differences in energy, calcium, and vitamin D intake among the three skater groups. All descriptive statistics are given as mean ± standard deviation (sd).

## Results

Table 1 describes the skaters’ demographic characteristics, mean energy, vitamin D, and calcium intakes. Of the 36 skaters, 10 were single, 8 were pair, and 18 were dancers. Their mean BMI mean was 19.8 ± 2.1, ranging from 15.1-23.3. Only 1 skater had a BMI that was classified as “underweight” using the CDC growth charts matched for age and gender. Mean % body fat for the skaters was 19.2 ± 5.8 but had a wide range of 7.3-31.2. Mean weekly training time was 18.25 ± 4.1 hours skating per week, with an additional 5.9 hours per week dedicated to other non-skating physical training activities. There were no significant differences in intakes of energy, vitamin D, calcium or training time among the skater types, however on average they were below recommended dietary intakes for their reference population [7]. Of the 36 skaters, only 5 skaters demonstrated intakes consistent with the reference norms; the remaining averaged 500 kcals below standard intakes. All skaters were below their estimated DRI for women with high physical activity levels. Similarly, only 1 skater met the DRI for vitamin D, all were below recommended intake, with an average deficit of 2.2 ± 2.6 mcg. Twelve of the 36 skaters had calcium intakes below their recommended intakes [8]. There were no significant differences in BMI or body fat % between the different skater disciplines.

**Table 1:** Means for demographic characteristics, dietary intake,and body composition of 36 elite skaters

**Characteristics**	**Mean (sd)**	**Range**
**Age (years)**	16 ± 2.5	13-22
**Weight (kg)**	48.5 ± 6.6	30.6-50.1
**Energy Intake (kcal)**		
**Daily *(reference normal)***^***7***^	1491.4 ± 471.2 *(1993 ± 45.7)*	565.8-2654.4
**Kcal/kg *(recommended intake)***^***8***^	31.8 ± 13.2 *(71)*	10.6-68.9
**Vitamin D (mcg)**	3.1 ± 2.6 *(5)*	0.2-10.8
**Daily *(recommended intake)***^**8**^		
**Calcium (mg)**	763.3 ± 438.1 *(793 ± 21.5)*	175-2466
**Daily (*reference normal*)**^**7**^		
**BMI**	19.8 ± 2.1	15.1-23.3
**Total BMD z score**	0.65 ± 0.89	-1.56 - 2.6
**Pelvic BMD z score**	2.02 ± 1.0	-0.25 - 3.68
**Spine BMD z score**	0.12 ± 0.82	-1.38 - 2.07
**Leg BMD z score**	1.25 ± 1.03	-1.22 - 3.84
**%Total Body Fat**	19.2 ± 5.8	7.3-31.2

**Figure 1:**
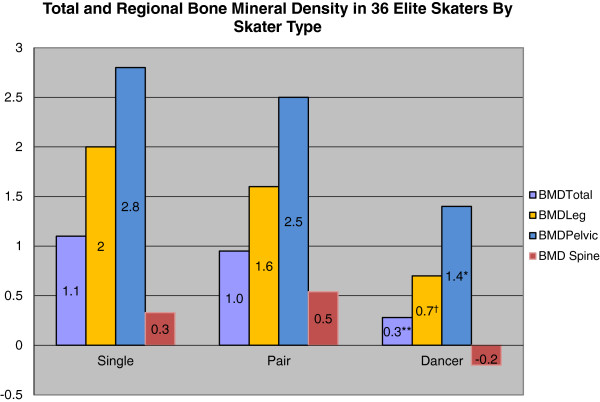
**Comparison of site specific bone mineral density z scores among skater type. **Significant differences by ANOVA: *p = 0.003 for Single and Pairs vs Dancer; **p < 0.001 Single vs Dancer; †p = 0.001 Single vs Dancer.

Average BMD z-scores were above mean reference norms for total body and all regions measured (Figure 1). There was a wide range BMD scores within each discipline for both total and regional measures as indicated by their standard deviations. Negative z-scores however were only seen for spine BMD, and no skater in any discipline had a z-score outside of 2 standard deviations of the mean.

### Predictors of bone mineral density

When controlling for all other variables, skater discipline (single, pair, or dancer) and BMI were the only significant predictors of total and all site-specific BMD regions measured in our model. Skaters with the lowest BMI had the lower BMD scores across all BMD regions measured except the pelvis. While there was no significant difference in BMI among the 3 skater disciplines, regression analysis showed that total BMD increased with increasing BMI in the total group of skaters (R = 0.60; p < 0.001). The effect of skater discipline on BMD variables is shown in Figure 1. Single and pair skaters each had higher z scores for total body BMD than did dancers. This was significant for single vs dancer skaters. Both single and pair skaters had significantly higher pelvic z scores than dancer skaters. Single skaters also had significantly higher leg z scores than dancer skaters. There was no significant difference in spine z-scores among the three groups.

## Discussion

The female athlete triad refers to the interrelationships among energy availability, menstrual function, and bone mineralization. If energy deficits are extreme, and body weight and fat mass are very low, estrogen levels fall, with delayed menarche in younger girls and menstrual irregularities. [9] Bone demineralization may ensue, particularly when intakes of vitamin D and calcium are insufficient, ultimately increasing stress fracture risk. Low energy intakes and suboptimal amounts of bone building nutrients have been reported in figure skaters [10-12]. The degree in which bone loading and physical training counterbalances the detrimental effects of poor nutrition on bone density in this unique group of athletes has not been studied. Furthermore, stratifying by skater discipline, as a proxy for the extent of mechanical loading experienced, has never been attempted, and is the greatest contribution of this study.

The Academy of Sports Medicine recommends that the WHO criteria (z-score of −2.0) be used for identifying risk of osteoporosis in adult female athletes [13]. Defining BMD z-scores cut-offs for predicting fracture risk in adolescents is more difficult, as there is insufficient data on how to adjust BMD for bone size, pre-pubertal age, and skeletal maturity in growing children. The International Society for Clinical Densitometry states that children with a total body BMD z-score of −2.0 (using a pediatric database matched for age and gender) are considered to have “low bone mineral density for chronological age”. [14] None of our skaters had z-scores less than (−)2.0, indicating that they were not at risk for osteoporosis by any of the established criteria for either adult or adolescent female athletes. Because BMD in female athletes in general is higher than sedentary controls, a more stringent cut-off is recommended by the American College of Sports Medicine [15]. Female athletes who have a history of nutritional deficiencies, stress fractures, or other clinical risk factors together with a “low” BMD z-scores (between −1.0 and −2.0 or greater) are considered to be at osteopenic risk. Suboptimal reported intakes of energy, vitamin D and calcium in our study are somewhat suggestive of a possible clinical deficiency. Even with this possibility, only two of the skaters qualify as at risk. No skater had a history of stress fractures.

Energy intakes for the skaters in this study were similar to those reported in other studies of figure skaters and lower than the 45 kcal/kg suggested for athletes who train for more than 90 minutes per day. [16] Some of this may be explained by underreporting. Intakes reported here were cross sectional in nature and only during training, when the skaters may have been monitoring their intakes carefully. They do not represent long term and usual intakes. In conjunction with this, mean BMI and percent body fat were relatively unremarkable for this group of skaters, and comparable to that reported in other groups of female athletes participating in weight bearing sports-although both variables ranged markedly among athletes. BMI in our group of skaters averaged 19.1 ± 2.1 compared to female athletes participating in basketball, volleyball, track, softball, soccer, and tennis which averages ranged between 21.6 ± 2.5 and 23.0 ± 2.4. Percent body fat in gymnasts and speed skaters was 13.1 ± 4.8 and 23.7 ± 7.3 compared to our skaters which averaged 20.2 ± 6.0 [17-21].

It is not surprising that we found a relationship between BMI and BMD z-score in our population. Increases in BMD typically correspond to increases in body size as indicated by weight, height or BMI, a phenomenon that is well recognized [22-24]. However, many athletes of low weight status, who participate in intense physical activity, can compensate for this effect. This may explain why some of our skaters with BMI’s below the norm for age as plotted on the CDC (2000) growth charts still demonstrated BMD scores > 100% above their age and weight matched norms. Therefore, even though our skaters showed a positive relationship between BMI and BMD, meaning those with the greatest BMI had a greater BMD, the BMD z scores of our skaters when compared to reference norms were still greater despite a lower BMI.

As might be predicted from what is known about the beneficial effects of jumping and other stressors on bone BMD, single and pair skaters did seem to be better protected from low total body BMD than dancer skaters, even after controlling for dietary intake variables, BMI, and % body fat. In the pelvic and leg regions, which receive the greatest impact of force (the pelvic and leg region), single skaters had significantly higher z-scores than dancer skaters. Pair skaters also had significantly greater pelvic z scores than their dancer counterparts. Since other factors were controlled for in this study, this finding is likely to relate to a training effect. This is also supported by the fact that there was no difference in spine bone density among the groups, which does not receive as much of the impact of landing, among the three skater disciplines.

Disagreement among measures of BMD taken by different DXA models, makes additional comparisons of our data to other reference norms difficult [23,25]. However, values for total BMD in our skaters were similar to that found in a group of intercollegiate female athletes participating in weight-bearing sports such as gymnastics, soccer, volleyball and track, who were measured on the same DXA unit and software package [22]. These healthy 20 female athletes had a similar BMI (average of 19.1 kg/m^2^), to our population. Their absolute BMD was 1.2 gm/cm^2^ compared to our group mean absolute BMD of 1.1 (range: 0.9-1.3) gm/cm^2^. Field hockey players were also studied using this system. Their absolute BMD was higher than our skaters, (1.3 ± 0.05), but they were older (mean age: 27 ± 3 and had a higher BMI of 22 ± 1.3), which may explain increased BMD over our smaller, younger study population. Absolute BMD measures in sedentary controls used for comparison in their study (but with a greater weight) were equivalent to our BMD, supporting again that physical activity in our skaters compensated for smaller body size [22,23].

In conclusion, our study shows that bone mineral density varies across skater discipline, with single skaters receiving the largest benefit from training effect in bone loading regions. Skater dancers may be at higher risk since their training does not compensate for the potential of low energy and bone building micronutrient availability as well as do the more intense exercise of the singles and pair dancers.

## Abbreviations

DXA: Dual Energy Photon Absorptiometry; BMD: Bone Mineral Density; TB: Total Body; L: Leg; S: Spine; P: Pelvis; gm/cm^2^: Grams Per Centimeter Squared; BMI: Body Mass Index; Sd: Standard Deviation.

## Competing interests

This work was supported in part by funds provided by the U.S. Department of Agriculture Cooperative State Research Education > Extension with grant #2006-35200-17259 and USDA Agricultural Research Service under agreement No 58 1950-7-707. Any opinions, findings, conclusions or recommendations expressed are those of the authors and do not reflect the view of the US Department of Agriculture. This study was also supported by a non-restricted grant to Tufts University from the Gerber Products Company.

## Authors’ contributions

KP, JD, and PZ drafted and revised the manuscript. JK reviewed the bone density data and confirmed its validity as well as general conclusions drawn from it. PZ conceived of the study and participated in its design and data collection. All authors read and approved the final manuscript

## References

[B1] Slemenda CW Johnston CC High intensity activities in young women: site specific bone mass effects among female figure skaters Bone Miner 1993 20 125 132 10.1016/S0169-6009(08)80021-9 8453328

[B2] Oleson CV Busconi BD Baran DT Bone density in competitive figure skaters Arch Phys Med Rehabil 2002 83 122 128 10.1053/apmr.2002.26246 11782842

[B3] Smith AD The young skater Clin Sports Med 2000 19 741 755 10.1016/S0278-5919(05)70235-0 11019738

[B4] Ziegler PJ Kannan S Jonnalagadda SS Krishnakumar A Taksali SE Nelson JA Dietary intake, body image perceptions, and weight concerns of female US International Synchronized Figure Skating Teams Int J Sport Nutr Exerc Metab 2005 15 550 566 16327035 10.1123/ijsnem.15.5.550

[B5] Greydanus DE Omar H Pratt HD The adolescent female athlete: current concepts and conundrums Pediatr Clin North Am 2010 57 697 718 10.1016/j.pcl.2010.02.005 20538152

[B6] Johannsen N Binkley T Englert V Neiderauer G Specker B Bone response to jumping is site-specific in children: a randomized trial Bone 2003 33 533 539 10.1016/S8756-3282(03)00220-5 14555256

[B7] Wright JD Wang CY Kennedy-Stephenson J Dietary intake of ten key nutrients for public health, United States: 1999-2000 Adv Data 2003 334 1 4 12743879

[B8] Position of the American Dietetic Association, Dietitians of Canada, and the American College of Sports Medicine Nutrition and Athletic Performance J Am Diet Assoc 2009 109 509 527 19278045 10.1016/j.jada.2009.01.005

[B9] Birch K Female Athlete Triad BMJ 2005 330 7485 244 246 10.1136/bmj.330.7485.244 15677660 PMC546077

[B10] Ziegler P Hensley S Roepke JB Whitaker SH Craig BW Drewnowski A Eating attitudes and energy intakes of female skaters Med Sci Sports Exerc 1998 30 583 586 10.1097/00005768-199804000-00017 9565941

[B11] Ziegler P Nelson JA Barratt-Fornell A Fiveash L Drewnowski A Energy and macronutrient intakes of elite figure skaters J Am Diet Assoc 2001 101 319 325 10.1016/S0002-8223(01)00083-9 11269611

[B12] Ziegler P Sharp R Hughes V Evans W Khoo CS Nutritional status of teenage female competitive figure skaters J Am Diet Assoc 2002 102 374 379 10.1016/S0002-8223(02)90086-6 11902370

[B13] Kanis JA Melton LJ 3rd Christiansen C Johnston CC Khaltaev N The diagnosis of osteoporosis J Bone Miner Res 1994 9 1137 1141 7976495 10.1002/jbmr.5650090802

[B14] Writing Group for the ISCD Position Development Conference Diagnosis of osteoporosis in men, premenopausal women, and children J Clin Densitom 2004 7 17 26 10.1385/JCD:7:1:17 14742884

[B15] Nattiv A Loucks AB Manore MM American College of Sports Medicine position stand. The female athlete triad Med Sci Sports Exerc 2007 39 1867 1882 10.1249/mss.0b013e318149f111 17909417

[B16] Economos CD Bortz SS Nelson ME Nutritional practices of elite athletes. practical recommendations Sports Med 1993 16 381 399 10.2165/00007256-199316060-00004 8303140

[B17] Greydanus DE Patel DR The female athlete. Before and beyond puberty Pediatr Clin North Am 2002 49 553 580 vi. 10.1016/S0031-3955(02)00005-6 12119865

[B18] Carbuhn AF Fernandez TE Bragg AF Green JS Crouse SF Sport and training influence bone and body composition in women collegiate athletes J Strength Cond Res 2010 24 1710 1717 10.1519/JSC.0b013e3181d09eb3 20453684

[B19] Hoch AZ Pajewski NM Moraski L Prevalence of the female athlete triad in high school athletes and sedentary students Clin J Sport Med 2009 19 421 428 10.1097/JSM.0b013e3181b8c136 19741317 PMC2848387

[B20] Webster BL Barr SI Body composition analysis of female adolescent athletes: comparing six regression equations Med Sci Sports Exerc 1993 25 648 653 8492694

[B21] Moon JR Eckerson JM Tobkin SE Estimating body fat in NCAA Division I female athletes: a five-compartment model validation of laboratory methods Eur J Appl Physiol 2009 105 119 130 10.1007/s00421-008-0881-9 18936958

[B22] Madsen KL Adams WC Van Loan MD Effects of physical activity, body weight and composition, and muscular strength on bone density in young women Med Sci Sports Exerc 1998 30 114 120 9475652 10.1097/00005768-199801000-00016

[B23] Sparling PB Snow TK Rosskopf LB O'Donnell EM Freedson PS Byrnes WC Bone mineral density and body composition of the United States Olympic women's field hockey team Br J Sports Med 1998 32 315 318 10.1136/bjsm.32.4.315 9865404 PMC1756126

[B24] Pettersson U Nordstrom P Alfredson H Henriksson-Larsen K Lorentzon R Effect of high impact activity on bone mass and size in adolescent females: A comparative study between two different types of sports Calcif Tissue Int 2000 67 207 214 10.1007/s002230001131 10954774

[B25] Soriano JM Ioannidou E Wang J Pencil-beam vs fan-beam dual-energy X-ray absorptiometry comparisons across four systems: body composition and bone mineral J Clin Densitom 2004 7 281 289 10.1385/JCD:7:3:281 15319498

